# Smartphone Restriction and Its Effect on Subjective Withdrawal Related Scores

**DOI:** 10.3389/fpsyg.2018.01444

**Published:** 2018-08-13

**Authors:** Tine A. Eide, Sarah H. Aarestad, Cecilie S. Andreassen, Robert M. Bilder, Ståle Pallesen

**Affiliations:** ^1^Department of Global Public Health and Primary Care, Faculty of Medicine, University of Bergen, Bergen, Norway; ^2^Department of Psychosocial Science, Faculty of Psychology, University of Bergen, Bergen, Norway; ^3^Department of Clinical Psychology, Faculty of Psychology, University of Bergen, Bergen, Norway; ^4^Department of Psychiatry and Biobehavioral Sciences, David Geffen School of Medicine, University of California, Los Angeles, Los Angeles, CA, United States

**Keywords:** smartphone, restriction, withdrawal, FoMO, experimental study, behavioural addiction, PANAS

## Abstract

Excessive smartphone use has been associated with a number of negative consequences for the individual and the environment. Some similarities can be observed between excessive smartphone usage and several behavioural addictions, and continual usage constitutes one of several characteristics included in addiction. In the extreme high end of the distribution of smartphone usage, smartphone restriction might be expected to elicit negative effects for individuals. These negative effects may be regarded as withdrawal symptoms traditionally associated with substance-related addictions. To address this timely issue, the present study examined scores on the Smartphone Withdrawal Scale (SWS), the Fear of Missing Out Scale (FoMOS) and the Positive and Negative Affect Schedule (PANAS) during 72 h of smartphone restriction. A sample of 127 participants (72.4% women), aged 18–48 years (*M* = 25.0, *SD* = 4.5), were randomly assigned into one of two conditions: a restricted condition (experimental group, *n* = 67) or a control condition (control group, *n* = 60). During the restriction period participants completed the aforementioned scales three times a day. The results revealed significantly higher scores on the SWS and FoMOS for participants allocated to the restricted condition than those assigned to the control condition. Overall the results suggest that smartphone restriction could cause withdrawal symptoms.

## Introduction

Modern mobile technologies have become increasingly popular and more advanced over the last decade. State of the art (i.e., smartphones) includes several multimedia functions, which enable users to be constantly connected and have access to an uninterrupted flow of real time data from social networking sites (SNSs; Valderrama, 2014; Chóliz et al., 2016). Consequently, the smartphone has become a crucial component in peoples’ lives, with 73% reporting that they would feel panic if they had misplaced their smartphone and 58% reporting checking it at least once every hour (Lookout, 2012).

Excessive and problematic smartphone usage, also referred to as an (behavioural) addiction (Andreassen et al., 2013; Billieux et al., 2015a), has potentially harmful effects (see Elhai et al., 2017, for a systematic review). Research indicates that overuse can lead to undesired outcomes for both the individual and their surroundings and may be of significant concern for public health (Tossell et al., 2015; van Deursen et al., 2015). Some studies indicate that excessive smartphone use can lead to musculoskeletal impairment (İnal et al., 2015; Xie et al., 2016), poor academic performance (Lepp et al., 2014), anxiety and depression (Demirci et al., 2015; Elhai et al., 2016) as well as poor sleep quality (Chang et al., 2015). The term behavioural addiction refers to an addiction that is non-chemical or non-substance related in nature and, that prior to Griffiths (1996) article, was often termed non-substance use addiction. Smartphone addiction has emerged as a subcategory of behavioural addictions. According to the component model of addiction Griffiths (2005) suggested that it was characterized by six components, these including salience, mood modification, tolerance, withdrawal symptoms, conflict, and relapse. These components were assumed to be common for both addictions related to substance use as well as for behavioural addiction. The *withdrawal symptoms* component refers to the unpleasant psychological and physiological effects that occur as a consequence of discontinuance of the particular activity. The dominating withdrawal effect may vary for each individual in terms of psychological and physiological outcomes. Psychological withdrawal symptoms refer to effects such as moodiness, irritability, and anxiousness, while physiological withdrawal symptoms include sweats, nausea, insomnia, headaches, and so on. Psychological withdrawal symptoms are effects that have been well documented in substance use addiction (Orford, 2001), and there is now a growing body of evidence also suggesting that withdrawal symptoms exist for behavioural addictions, such as pathological gambling (Griffiths, 2004).

So far the number of studies that have focused on the effects of restricting access to smartphones is limited. One study revealed that restriction made participants significantly more anxious over time (Cheever et al., 2014). However, this effect was found only in individuals who were heavy or moderate users of smartphones (Cheever et al., 2014). In another study being unable to answer incoming calls on ones’ smartphone was found to cause increased heart rate and blood pressure, as well as feelings of anxiety and unpleasantness (Clayton et al., 2015). Several other studies have examined smartphone restriction and potential addiction through various designs (Sapacz et al., 2016; Cutino and Nees, 2017; van den Eijnden et al., 2017). These findings suggest that withdrawal symptoms may be in play when people’s access to their mobile phone is restricted. A phenomenon that may explain symptoms of smartphone restriction withdrawal is fear of missing out (FoMO), which denotes an overhanging concern that one is excluded from taking part in or sharing enjoyable experiences others might be having (Przybylski et al., 2013). Online participation might be particularly attractive due to the immediate access to information about friends and events, where individuals high in FoMO might gravitate toward these social media channels. Furthermore, restriction from access to these channels might provoke withdrawal related symptoms. Several studies attest to a positive association between FoMO and continual excessive smartphone use (Rosen et al., 2013a,b; Lepp et al., 2014; Clayton et al., 2015; Elhai et al., 2016; Fuster et al., 2017). In line with this, a growing body of research on excessive smartphone use has shown it to be strongly associated with addictive use of online social media (Andreassen et al., 2013, 2016; Salehan and Negahban, 2013; Fuster et al., 2017; Lopez-Fernandez et al., 2017). Characteristics of the smartphone, such as size and portability, could facilitate multiple reinforcement pairings associated with the stimuli, which rapidly may instigate an addictive behavioural pattern. There exist different viewpoints regarding the addiction to technology, whether they involve being addicted to the medium itself or whether the medium is merely a promoter of other addictions. There are three main views regarding this issue: (1) one can be addicted to the medium itself; (2) one could be addicted to the medium, because it grants access to different types of content that is accessible only through the medium; and (3) one is only addicted to the content the medium makes accessible and not to the medium itself. Young (1998) argues that the medium is what causes addiction due to the fact that the content would not be accessible without it, while Griffiths et al. (2016) argue that the medium itself is not addictive, but the medium is used as a platform/source that promotes addictions. Nevertheless, some findings from case studies have indicated that a few individuals seem to be addicted to the Internet itself. These individuals often use the Internet for chat rooms and activities that are accessible only through the Internet (Griffiths et al., 2016). This argument has also been used to describe people that seem to be addicted to social media and SNSs (Kuss and Griffiths, 2011; Griffiths et al., 2014). In addition, there is some debate to whether one can go as far as to call excessive or problematic smartphone usage, addiction (Billieux et al., 2015b). Independently of this discussion, there exists some resemblance between excessive smartphone use and behavioural addiction, which makes an investigation of the potential withdrawal symptoms upon restriction of interest.

When considering withdrawal symptoms, the physiological are more specific to the substance use-related addictions (Shiffman and Jarvik, 1976; Griffiths, 1996, 2005; Etter, 2005), whereas withdrawal symptoms in behavioural addictions typically consist mainly of psychological symptoms (Griffiths, 1996, 2005; Alavi et al., 2012; Parlak and Eckhardt, 2014). Several studies have used anxiety measures and the related negative effects as means for investigating individual experience during restriction periods in persons suffering from different behavioural addictions (Przybylski et al., 2013; Cheever et al., 2014; Clayton et al., 2015). However, there is little research on withdrawal in behavioural addiction (Starcevic, 2016).

Studies of substance addiction withdrawal have shown that there are certain temporal trends regarding symptom development. Knowledge about these effects can be highly useful as the issue of withdrawal symptoms in behavioural addictions has yet to be sufficiently researched. Shiffman and Jarvik (1976) studied smokers who abstained from cigarettes over a certain period. The results indicated that the symptoms had a U-shaped function, whereby the symptoms were more salient in the beginning and toward the end of a restriction period. However, a study on alcohol withdrawal found the symptoms to follow an inverted U-curve (Sellers and Kalant, 1976). These findings indicate that there might be some differences across various addictions regarding the temporal shape of the withdrawal symptoms. In addition, Hughes et al. (2004) performed a systematic literature review where they studied smokers, and found that most of the relapses happened within the first 8 days. Thus, it could be argued that there should be greater clinical focus on the first week of restriction periods (Hughes et al., 2004). There is little research done on withdrawal and its temporal development in behavioural addiction.

Against this backdrop, we designed an experiment comparing 72 h of smartphone restriction to a control condition with no restriction. We hypothesised that participants in the experimental condition would score significantly higher on smartphone withdrawal symptoms, fear of missing out and negative mood, albeit lower on positive mood, compared to controls (H1), reflecting main effects of condition. We also expected that negative symptoms would be larger in the beginning of the registration period compared to later (H2), reflecting main effects of time. Finally, we expected a larger drop in withdrawal symptoms over time in the experimental than in the control condition (H3), which would be reflected by significant two-way interaction (Condition × Time) effects.

## Materials and Methods

### Participants

The sample comprised 127 participants, 72.4% women (*n* = 92) and 27.6% men (*n* = 35). All participants were between the ages of 18 and 48 years old, with a mean age of 25 years (*SD* = 4.5). In all, 79.5% (*n* = 101) were full-time students attending higher education in Bergen.

### Instruments

#### Demography

The participants were asked to complete items regarding their age, gender, relationship status, and student status.

#### Smartphone Frequency and Use Items

The questionnaire consisted of five items where the participants rated themselves on topics such as frequency, duration and characteristics (e.g., “Do you use your smartphone every day?”) of smartphone use. The questionnaire is reproduced in Appendix A.

#### Smartphone Withdrawal Scale (SWS)

This scale was included in the study for measuring the degree of withdrawal symptoms related to smartphone restriction. The Smartphone Withdrawal Scale (SWS) is a modified version of the Cigarette Withdrawal Scale (CWS; Etter, 2005). Although cigarette withdrawal concerns a substance, there is a substantial overlap between symptoms of tobacco withdrawal and withdrawal symptoms associated with behavioural addiction (American Psychiatric Association, 2013). The CWS originally consists of 21 items divided into six subscales (Depression-Anxiety, Craving, Irritability-Impatience, Difficulty Concentrating, Appetite-Weight Gain, and Insomnia), but in the present study the Appetite-Weight Gain and the Insomnia subscale were not included as they seemed less relevant for smartphone withdrawal. Four items on the Craving subscale, specific to cigarette use, were modified to become relevant for smartphone withdrawal. In addition, the scale was altered from a trait to state format, by wording the questions from a general to a specific state (e.g., “The only thing I can think about in this moment, is my smartphone”; see Supplementary Material for a full list of items). The modified scale consists of 15 items rated on a five-point Likert scale ranging from 1 (*totally disagree*) to 5 (*totally agree*). A composite score was calculated based on the sum score of all the 15 items. The Cronbach’s alpha for the SWS was shown to be very good across all the nine times it was measured, ranging from 0.88 to 0.92.

#### Positive and Negative Affect Schedule (PANAS)

The Positive and Negative Affect Schedule (PANAS) (Watson et al., 1988) was used to measure self-reported mood and consists of 20 items, 10 items related to the Positive Affect Schedule (PA) and 10 items related to the Negative Affect Schedule (NA). These items describe different affective states, such as *hostile* and *excited*. The participants scored each item on a five-point Likert scale from (*very slightly or not at all*) to 5 (*extremely*), based on their present state. In the present study, the Cronbach’s alpha reliabilities for both the PA (0.87–0.92) and the NA (0.77–0.85) subscale were shown to be good to excellent across the nine times of measuring.

#### Fear of Missing Out Scale (FoMOS)

The Fear of Missing Out Scale (FoMOS) (Przybylski et al., 2013) was used as a self-reported measure of FoMO. However, in the present study, the scale was adapted into a state measure by wording the questions from a general to a specific and present state. The scale consists of 10 items (e.g., “I fear others have more rewarding experiences than me right now”) rated on a five-point Likert scale from 1 (*not at all true of me*) to 5 (*extremely true of me*). The FoMOS demonstrated good internal consistency across the nine times of measuring with an alpha reliability ranging from 0.80 to 0.87.

The measures used to characterise smartphone usage were administered one time, while the battery of withdrawal related scales was completed at nine intervals during the restriction period. These withdrawal-related scales comprised the dependent variables. Time represented the repeated measures for each participant (nine times), which enabled an investigation of intra-individual variations. Condition represented either restricted or control.

### Procedure

The participants were recruited through advertisement on Facebook and by personal appeal. Participants who did not use their smartphone for at least 1 h on a daily basis were excluded. The study took place over ten weekends during the period from October 2016 to February 2017. Each participant was assigned a unique ID and randomised into either a restricted or a control condition by an online randomiser calculator (Urbaniak and Plous, 2015).

**FIGURE 1:**
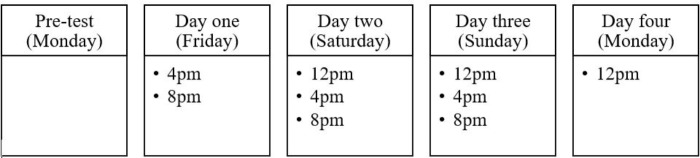
Progression model illustrating the experimental design.

**FIGURE 2:**
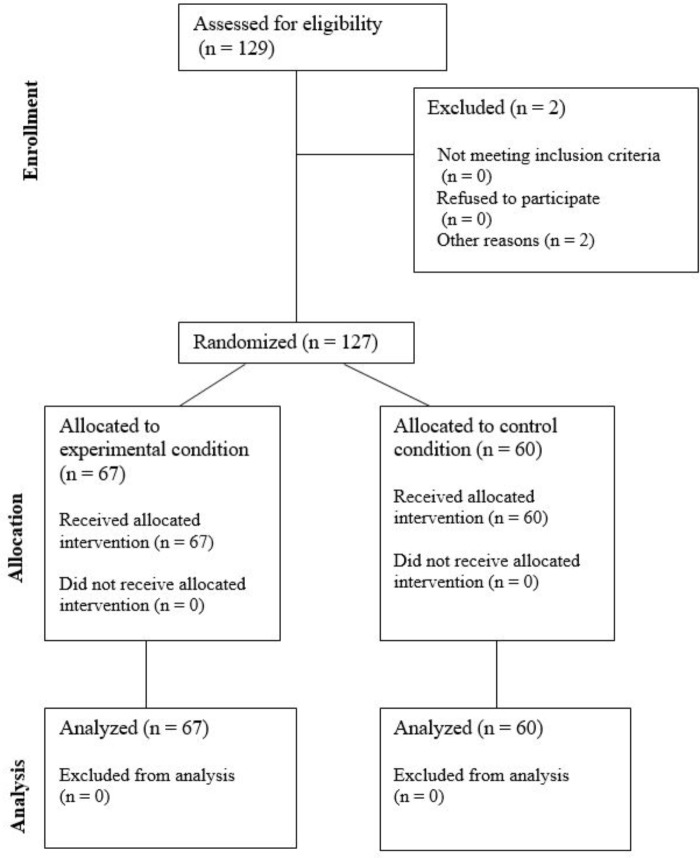
Flowchart participant recruitment.

The Monday before the experimental weekend (Friday–Monday; see **Figure 1**) the participants received an email containing a link to a web-based survey (demographics and smartphone usage). Upon inclusion, all participants were given a unique, consecutively allocated id-number and randomly divided into either restricted or control condition (see **Figure 2**). On Friday, those allocated to the restricted condition (experimental group; *n* = 67) were instructed to turn off their smartphones and hand them in. The smartphone was placed in a secure locked cabinet over the weekend. Those allocated to the control condition (control group; *n* = 60) were allowed to keep and use their smartphone as usual. During the restriction period (72 h), the participants were instructed to complete relevant questionnaires (SWS, FoMOS, and PANAS) three times a day in a pamphlet they received on the first experimental day. On the following Monday, the participants handed in the completed questionnaires. Those in the restricted condition got their smartphones back and responded to an open-ended qualitative question regarding challenges related to the restriction period. All participants received a remuneration of 500 NOK for taking part in the study. The amount was undisclosed in advance to ensure the primary motivation for participation in the study.

### Ethics

The study was conducted in accordance to the Declaration of Helsinki and approved by the Norwegian Data Protection Authority (project no. 49769) and the ethics committee consisted of one person, Belinda Gloppen Helle from the Norwegian Centre for Research Data. All participants were recruited from the general adult population (at least 18 years old) and all gave electronic informed consent.

### Data Analysis

A linear mixed model analysis was applied and a restricted maximum likelihood approach was used as this produces unbiased estimates of variance and covariance parameters. Random intercept was included in the models (Harville, 1977; West et al., 2014). In the analysis, between-subjects factors reflected the potential difference between the individuals in the restricted condition and the control condition, in terms of smartphone withdrawal (determined from the SWS score), fear of missing out (determined from the FoMOS score), and positive/negative affect (determined from the PANAS scores). A power-analysis showed that the number of participants included would be sufficient for a power of 0.80 in the cases of medium effect sizes for the fixed factors and an expected correlation coefficient between repeated measures of 0.5 (Hedeker et al., 1999). All analyses were conducted using SPSS Version 23.

On the items from the SWS scale, completed during the restriction period, the missing data comprised 4.4% of the total. FoMO items had 4.2%, the PA scale 4.5%, and the NA scale had 4.2% missing data. However, the linear mixed model analytic approach makes it possible to use the available data for units where time points are missing.

## Results

The dataset will be made available upon request to the TE.

### Descriptives

Smartphone usage was measured prior to the experimental weekend. The difference in self-reported smartphone usage did not differ between groups (*t* = 1.36, *df* = 125, *p* = 0.177). See **Table 1** for more detailed descriptives. There was no difference in gender distribution (χ^2^= 0.373, *df* = 1, *p* = 0.541) between the two conditions.

**Table 1:** Mean (M) and standard deviations (SD) of reported smartphone usage and experienced difficulty during the smartphone restriction period in percentage.

	*M* (*SD*)	Percentage
**Reported usage for**		
Restricted group	2.79 (0.85)	
Control	2.62 (0.56)	
**Challenges in restriction period related to**		
Process apps		49.3%
Social communication		49.3%
Inaccessibility		43.3%
Planning		40.3%
Alarm/clock		32.8%
Music/podcast		25.4%
Social networking apps		13.4%
Security		10.4%
Pass time		6.0%


*Smartphone usage value of three indicate usage of 3 to 6 h.*

### Analysis of experiment

#### The Effect of Smartphone Restriction on Withdrawal Symptoms (see **Tables 2, 3**)

**Table 2:** The effect of smartphone restriction on withdrawal (SWS) scores by linear mixed models.

Time	Estimate	Standard error	*t*	*F*
1	0.177	0.071	2.48*	
2	0.133	0.072	1.85	
3	0.026	0.072	0.359	
4	0.053	0.071	0.745	
5	-0.050	0.072	-0.696	
6	-0.011	0.072	-0.150	
7	0.032	0.072	0.449	
8	0.047	0.071	0.657	
9				
Condition				4.90*
Time				2.83**
Condition^∗^time				0.226


*Time 9 represents the reference time. SWS, Smartphone Withdrawal Scale. ^∗^p < 0.05, ^∗∗^p < 0.01, ^∗∗∗^p < 0.005, ^∗∗∗∗^p < 0.001.*

**Table 3:** The mean and standard deviation for each condition on the SWS, FoMOS, and PANAS at Time 1–9.

	Restricted				Non-restricted			
Time	SWS	FoMO	PA	NA	SWS	FoMO	PA	NA
1	1.69 (0.647)	2.01 (0.720)	2.77 (0.713)	1.34 (0.392)	1.57 (0.655)	1.86 (0.558)	2.78 (0.737)	1.27 (0.367)
2	1.68 (0.660)	2.05 (0.744)	2.61 (0.576)	1.32 (0.422)	1.53 (0.562)	1.76 (0.642)	2.67 (0.854)	1.29 (0.405)
3	1.57 (0.561)	1.88 (0.793)	2.63 (0.719)	1.32 (0.394)	1.40 (0.552)	1.75 (0.624)	2.79 (0.829)	1.26 (0.389)
4	1.60 (0.650)	1.93 (0.754)	2.61 (0.820)	1.34 (0.471)	1.44 (0.556)	1.77 (0.631)	2.73 (0.791)	1.20 (0.287)
5	1.57 (0.683)	1.87 (0.660)	2.53 (0.699)	1.27 (0.382)	1.32 (0.395)	1.68 (0.597)	2.63 (0.775)	1.18 (0.282)
6	1.54 (0.536)	1.81 (0.695)	2.47 (0.852)	1.27 (0.421)	1.37 (0.420)	1.59 (0.555)	2.71 (0.856)	1.24 (0.360)
7	1.62 (0.576)	1.86 (0.623)	2.30 (0.749)	1.33 (0.387)	1.41 (0.528)	1.64 (0.517)	2.60 (0.743)	1.25 (0.335)
8	1.65 (0.676)	1.85 (0.682)	2.43 (0.695)	1.31 (0.388)	1.43 (0.461)	1.60 (0.586)	2.57 (0.775)	1.21 (0.352)
9	1.53 (0.536)	1.74 (0.573)	2.57 (0.665)	1.21 (0.370)	1.36 (0.506)	1.62 (0.573)	2.64 (0.787)	1.19 (0.351)


**FIGURE 3:**
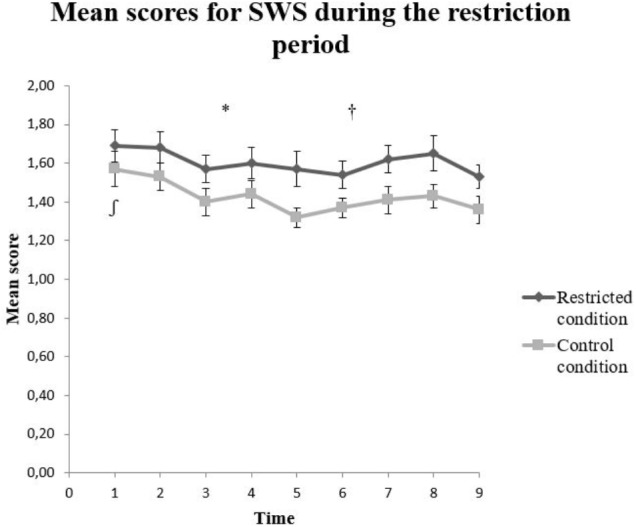
Mean scores on the Smartphone Withdrawal Scale (SWS) for the restricted and control condition. The error bars represent standard error mean for each value. ^∗^*p* < 0.05 for main effect of condition, ^†^*p* < 0.05 for main effect of time, and ^∫^*p* < 0.05 for Time 1 compare to Time 9.

On the SWS there was a statistically significant main effect of condition, *F*(1,124.97) = 4.90, *p* < 0.05, and time, *F*(8,951.19) = 2.83, *p* < 0.005 on the total score. The interaction effect between condition and time was not statistically significant, *F*(8,951.19) = 0.226, *p* = 0.986 (**Figure 3**). Specifically, Time 1 had a statistically significant higher SWS score compared to Time 9 (*t* = 2.48, *p* < 0.05) which represented the reference time.

#### The Effect of Smartphone Restriction on Fear of Missing Out (see **Tables 3, 4**)

**Table 4:** The effect of smartphone restriction on fear of missing out (FoMOS) scores by linear mixed models.

Time	Estimate	Standard error	*t*	*F*
1	0.239	0.064	3.72****	
2	0.149	0.065	2.28*	
3	0.114	0.065	1.75	
4	0.140	0.064	2.18*	
5	0.072	0.065	1.11	
6	-0.021	0.065	-0.328	
7	0.018	0.065	0.280	
8	-0.026	0.064	-0.407	
9				
Condition				3.99*
Time				8.17****
Condition^∗^time				0.652


*Time 9 represents the reference time. FoMOS, Fear of Missing Out Scale. ^∗^p < 0.05, ^∗∗^p < 0.01, ^∗∗∗^p < 0.005, ^∗∗∗∗^p < 0.001.*

**FIGURE 4:**
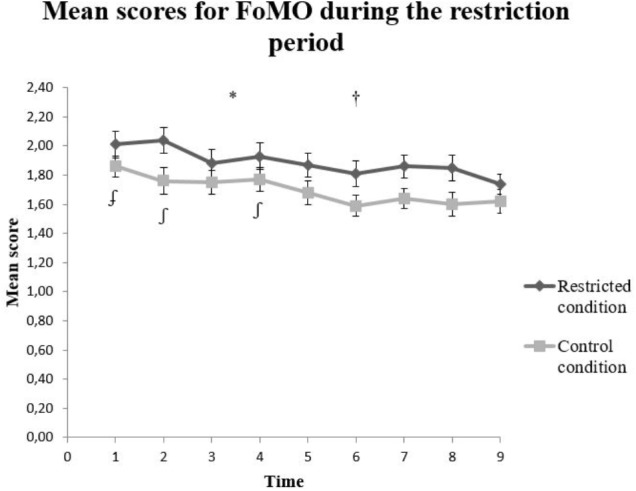
Mean scores on the Fear of Missing Out (FOMO) Scale for the restricted (*n* = 67) and control (*n* = 60) condition. The error bars represent standard error mean for each value. ^∗^*p* < 0.05 for main effect of condition, ^†^*p* textitp < 0.05 for effect of time, ^∫^*p* < 0.05 for Time 2 and Time 4 compared to Time 9, and ^∫^*p* < 0.001 for Time 1 compared to Time 9.

There was statistically significant main effect of condition, *F*(1,124.81) = 3.99, *p* < 0.05, and time, *F*(8,952.40) = 8.17, *p* < 0.001, on the total score of FoMOS. The interaction effect between condition and time was not statistically significant, *F*(8,952.40) = 0.652, *p* = 0.734 (**Figure 4**). Further, Time 1 (*t* = 3.72, *p* < 0.001), Time 2 (*t* = 2.28, *p* < 0.05), and Time 4 (*t* = 2.18, *p* < 0.05) had a statistically significant higher FoMOS score compared to the reference time (Time 9).

#### The Effect of Smartphone Restriction on Positive and Negative Affect (see **Tables 3, 5**)

**Table 5:** The effect of smartphone restriction on positive affect (PANAS) scores by linear mixed models.

Time	Estimate	Standard error	*t*	*F*
1	0.190	0.109	1.75	
2	0.101	0.111	0.914	
3	0.181	0.111	1.64	
4	0.045	0.110	0.405	
5	0.131	0.110	1.19	
6	0.002	0.110	0.015	
7	0.017	0.109	-0.155	
8	-0.017	0.109	-0.155	
9				
Condition				1.89
Time				3.72****
Condition^∗^time				0.865


*Time 9 represents the reference time. PANAS, Positive and Negative Affect Schedule. ^∗^p < 0.05, ^∗∗^p < 0.01, ^∗∗∗^p < 0.005, ^∗∗∗∗^p < 0.001.*

**FIGURE 5:**
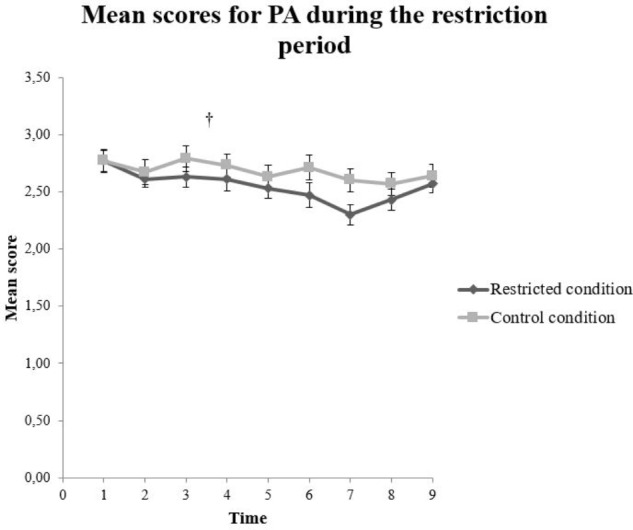
Mean scores on Positive Affect (PA) for the restricted (*n =* 67) and control (*n =* 60) condition. The error bars represent standard error mean for each value. ^†^*p* < 0.001 for main effect of time.

**FIGURE 6:**
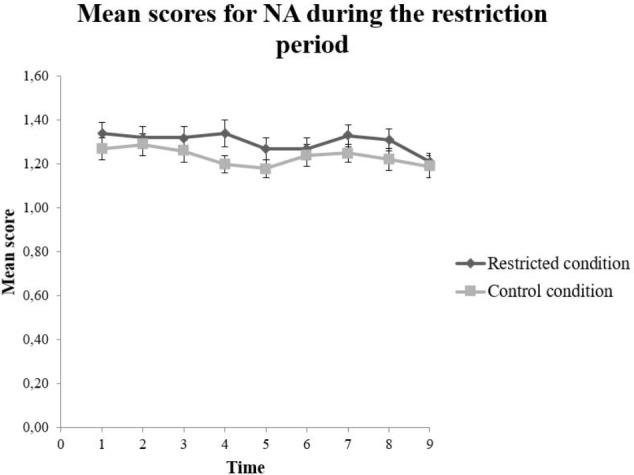
Mean scores on Negative Affect (NA) for the restricted (*n =* 67) and control (*n =* 60) condition. The error bars represent standard error mean for each value.

There was no statistically significant main effect for condition, *F*(1,125.15) = 1.89, *p* = 0.171 on PA. However, the analysis revealed a statistically significant main effect for time, *F*(8,951.23) = 3.72, *p* < 0.001, on the total score of PA. No significant results were found between each timepoint in the follow-up test. The interaction effect between condition and time on the PA score, *F*(8,951.23) = 0.865, *p* = 0.546, was not statistically significant (**Figure 5**). The NA score had no significant main effect for condition, *F*(1,124.23) = 1.73, *p* = 0.191, nor for time *F*(8,952.48) = 1.95, *p* = 0.050 (**Table 6**). Furthermore, the interaction effect between condition and time on the NA score *F*(8,952.48) = 0.730, *p* = 0.665, was not statistically significant (**Figure 6**).

**Table 6:** The effect of smartphone restriction on negative affect (PANAS) scores by linear mixed models.

Time	Estimate	Standard error	*t*	*F*
1	0.054	0.049	1.10	
2	0.069	0.049	1.40	
3	0.042	0.049	0.861	
4	-0.012	0.049	-0.252	
5	-0.030	0.049	-0.614	
6	0.028	0.049	0.570	
7	0.032	0.049	0.652	
8	0.000	0.049	0.003	
9				
Condition				1.73
Time				1.95*
Condition^∗^time				0.730


*Time 9 represents the reference time. PANAS, Positive and Negative Affect Schedule. ^∗^p < 0.05, ^∗∗^p < 0.01, ^∗∗∗^p < 0.005, ^∗∗∗∗^p < 0.001.*

## Discussion

The main aim of the present study was to investigate withdrawal symptoms, fear of missing out, and positive and negative affect associated with smartphone restriction over time. Based on the research design, the present study represents one of the first experimental studies undertaken on this topic. The findings were consistent with one of the hypotheses and previous research, with results showing that smartphone restriction contributed significantly to the explained variance symptoms of withdrawal and FoMO. However, restriction was not related with positive or negative affect.

There was a significant main effect for condition on the SWS where the restricted condition had a higher mean score compared to the control condition. More specifically, this evidence indicates that smartphone restriction provokes psychological withdrawal symptoms similar to those found in other behavioural addictions. The results also revealed a significant main effect for condition on FoMOS indicating that the FoMOS scores were significantly higher for the restricted condition, compared to the control condition, irrespective of the effect of time. FoMOS could be a representation of the social aspect of withdrawal and could therefore provide support for this hypothesis. These results could arise from a restriction of the immediate access to social networks, which provoke these negative effects. There was no significant main effect for condition on PA, thereby representing no significant difference between the restricted condition and the control condition in terms of PA scores. This indicates that being restricted from the smartphone does not cause a decrease in PA. Regarding NA, there was no significant main effect for condition. This result suggests that being restricted from the smartphone does not cause an increase in NA. These findings provide partial support to H1 by indicating that individuals are negatively affected when restricted from interacting with their smartphones.

A significant main effect of time for the SWS, FoMOS, and PA was found, indicating that the scores differed significantly over time, regardless of condition. Further, the main effect of time for NA was not significant. Hence, H2 was partially supported by the data. There was no significant interaction effect for the outcome variables (SWS, FoMOS, PA, and NA), which results in lack of support for H3. Consequently, the present study could not identify trends regarding the negative effects caused by the restriction period.

The reported negative effects (SWS and FoMOS) caused by an inability to interact with one’s smartphone could be linked to higher levels of stress (Lazarus and Folkman, 1984; Folkman, 2008) as some studies have suggested that using one’s smartphone can cause a temporary outlet for stress (Lavoie and Pychyl, 2001; Thomée et al., 2007). A study by Patel et al. (2006) revealed that children who played a handheld video game before surgery had lower levels of stress and anxiety than children who only had their parents present. A handheld video game does have some characteristics similar to smartphones, which makes this comparison relevant regarding the interpretation of the present findings. Although several games are available via smartphones, there are also some notably differences between video games and smartphones which put limits on the comparative effects. However, when in possession, a smartphone is immediately accessible with all the different process- and social apps. One can speculate that young adults may experience the same negative reinforcing effect of a smartphone in various daily stressful situations. If so, one may further argue that restriction of the same types of devices could restrict the negative reinforcing effect of the smartphone. However, these are merely speculations and further studies are needed to examine the possibilities of such a connection. From the PANAS scale, Negative Affect has been proven to be positively correlated with self-reported stress (Watson et al., 1988).

Another explanation for the findings regarding H1 can be linked to connectedness and an extension of the self. SNSs popularity have continued to grow since they first where introduced and they have developed to incorporate functions, such as instant messaging. It has been suggested that a possible explanation for SNSs being as popular as they have become is due to being able to connect to fundamental human needs. SNSs are able to offer their users social support by offering a way of being constantly connected to family, friends, and acquaintances 24/7. In addition, these instant message applications offer a private forum for peers to interact without supervision from others. This could help explain the high engagement users show to SNSs (Carbonell and Panova, 2017; Kuss and Griffiths, 2017). Smartphones have made it easier to access SNSs and therefore by restricting smartphone interaction one makes it more challenging to be constantly connected and fully engaged in the aspects of society facilitated by smartphones.

Another highly related term in regard to the social aspect of restriction is the extended self, proposed by Belk (1988). In the construct of the sense of self, he claims that an individual’s possessions represent an important part in reflecting one’s identities. When their possessions are taken away, a diminished sense of self would occur. This implies an emergence of negative emotions. One consequence of technological changes is the extension of the self into graphical representations of the individual, such as avatars that can affect our offline sense of self. The digital platform has gone from being somewhat private to becoming the main platform for revealing and projecting ourselves. An increase in sharing of private information on SNSs may leave the user in a vulnerable position, where frequent posts are required in order to maintain or gain control (Thompson and Cupples, 2008).

Being unable to ask questions, provide instructions or exchange personal information on the go could explain the higher score on the SWS and FoMOS. In addition, it could be related to the process apps that are accessible on the smartphone, which enables interaction with the general society through news, bus tickets, emails, and so on. This is in conformity with some of the challenges reported by the restricted participants, where almost half reported difficulty with being restricted from the process apps, as well as social communication. Further, the participants reported challenges associated with planning and immediate inaccessibility to other persons. The extended self provides an interesting view regarding use of technology. Through digital technology, the offline and online self becomes jointly constructed; thus, imposing a restriction on an individual that removes him/her from the online self, such as smartphone restriction, could provoke withdrawal related symptoms (Belk, 1988, 2013).

This study is one of the first investigating the effect of restriction of smartphone for the extension of time and by physically removing the smartphone. Few other studies have examined smartphone restriction, but with various designs. A study by Cheever et al. (2014), the participants were randomly assigned into one of two conditions: one condition turned in their smartphone, while the other condition was allowed to keep their smartphone but had to turn it off for the duration of the study. The experimental phase lasted for only 75 min. A second study investigated smartphone restriction for 3 h at a festival (van den Eijnden et al., 2017). In this study, the participants got to keep their smartphone, but had to put it in flight mode and the screen was made invisible by a seal. Regarding withdrawal trends, the former is the only one including trends. This is, however, difficult to compared with the present study due to the difference in duration.

### Strengths and Limitations

The dependent variables were included to assess different and relevant aspects of smartphone withdrawal and represent one key strength of the present study. The 72 h experimental phase, considerably longer than a previous smartphone restriction experiment allowed for detailed assessment of fluctuations in the dependent variable and is another asset of the present study (Cheever et al., 2014; van den Eijnden et al., 2017). The fact that the participants in the experimental condition handed in their smartphones during the restriction period ensured the integrity of the experiment.

In terms of limitations selection bias is a possible weakness of the present study as one can assume that individuals who were excessive users were less likely to participate. The participants could also pick freely the weekend they wished to participate. This could be a limitation considering that participants could adjust their weekend plans accordingly. A preponderance of females in the sample represents another limitation, as some studies have suggested that men and women engage in different types of smartphone usage. It is further conceivable that the participants used SNSs on other technological devices (e.g., laptop, tablet) during the restriction period. This should thus be controlled for in future studies. It could be argued that the present study did not imply real restriction as the participants could use other electronic devices by which they could access internet. However, as most people today use their mobile phones to access internet in situations when they have not access to a PC/tablet the present study did imply restriction regarding those types of situations. Also, it should be noted that some applications are only available on mobile phones. In addition, it should be kept in mind that the aim of the present study was to investigate mobile phone withdrawal specifically, and not internet withdrawal in general. The fact that the experimental group had higher scores on several withdrawal measures compared to the control group does also suggest that real restriction did occur. One of the scales used to measure smartphone withdrawal (SWS) was a modified cigarette withdrawal scale. Although the SWS had a high internal consistency it has not been used in any other studies, which may be regarded as a weakness. Further, the fundamental difference between smartphone and nicotine addictive properties is worth mentioning. In addition, a lack of baseline scores for the withdrawal related scores serves as another limitation for the present study. Finally, it should be noted that the difference between the experimental group and the control group in frequency of smartphone usage prior to the start of the restriction period could potentially be a limitation.

### Implications

In terms of behavioural addictions, the findings complement the body of evidence indicating that excessive smartphone usage embodies elements of addiction. Findings from the present study will aid the expansion of knowledge and understanding surrounding this part of the addiction field, such as the negative effects following restriction. These results actualize the focus on effects related to withdrawal in behaviours vulnerable to excessive use. Further, this study could aid future studies that will examine withdrawal related symptoms following restriction, as both strengths and weaknesses have been highlighted.

## Conclusion

The present study revealed that being restricted from ones’ smartphone increases withdrawal symptoms and fear of missing out, but do not influence positive and negative affect, specifically. The results indicate that a large part of the negative effects experienced by the participants in the restriction period, are similar to those of other types of behavioural addictions. In addition, the study included the time component in order to examine withdrawal trends, but the results was not significant. Given the result of the present study, it is important in future studies to fully explore the concept of smartphone addiction with focus on withdrawal symptoms. It would also be of interest to compare withdrawal trends across the spectrum of addictions. This is the first study of its kind to the authors’ knowledge, regarding complexity of design. Future studies should take strengths and limitations into account when investigating this topic further.

## Author Contributions

TE, SA, and SP conceived and designed the experiment and analyzed the data. TE and SA performed the experiments. TE, SA, SP, CA, and RB wrote the paper.

## Conflict of Interest Statement

The authors declare that the research was conducted in the absence of any commercial or financial relationships that could be construed as a potential conflict of interest.
