# Supplementary material for: Smartphone Restriction and Its Effect on Subjective Withdrawal Related Scores
Source: Front Psychol. 2018 Aug 13;9:1444. doi: 10.3389/fpsyg.2018.01444 (PMC6099124; doi:10.3389/fpsyg.2018.01444)
Supplement: Supplementary file 1 [file Data_Sheet_1.PDF]

1. Do you use your smartphone every day?

Yes

No

2. Please estimate the average number of hours you spend each day using your smartphone:

Less than 1 hour

1-2

3-6

7-10

11-15

16-20

More than 20 hours

3. Please estimate the average number of hours you spend each week using your smartphone:

1 hour or less

2-4

5-7

7-10

11-15

16-20

21-25

26-30

31-35

36-40

More than 40 hours

4. How long do you typically spend on your smartphone when you are using it?

2 minutes or less

3-5 minutes

6-9 minutes

10-14 minutes

15-19 minutes

20-29 minutes
30-44 minutes
45-59 minutes
60-89 minutes
90 minutes to 2 hours
2-4 hours
More than 4 hours

5. Please estimate the proportion of time spent using your smartphone that is for business purposes:
Zero
Less than 10%
10% - 20%
Approximately a quarter of the time (25%)
Approximately half the time (50%)
Approximately three-quarters of the time (75%)
75% - 95%
Almost always (95% - 100%)

6. The duration of my smartphone use is (please choose the best answer):
fairly consistent
varies
widely varies

7. How long I use my smartphone at any given time depends on (select all that apply):
the particular activity
my mood
how much time I have available
whether I am alone or with others

8. I use my smartphone to access the following activities (select all that apply):
apps or applications
books
camera or camcorder

driving directions or GPS navigation

emails

games

Internet

movies, TV shows and videos

music

news

pornography

social networking (such as Facebook or Twitter)

text messages

voice calls

other (please specify)
