# Supplementary material for: Smartphone Restriction and Its Effect on Subjective Withdrawal Related Scores
Source: Front Psychol. 2018 Aug 13;9:1444. doi: 10.3389/fpsyg.2018.01444 (PMC6099124; doi:10.3389/fpsyg.2018.01444)
Supplement: Supplementary file 2 [file Data_Sheet_2.pdf]

Appendix

Appendix B - Modified Smartphone Withdrawal Scale (SWS)

SWS

Instructions: Consider the following sentences by using the scale from 1 (strongly disagree) to 5 (strongly agree). Respond to all the sentences.

		**1 = Strongly disagre**				**5 = Strongly agree**
		**1**	**2**	**3**	**4**	**5**
1.	I feel depressed right now	❑	❑	❑	❑	❑
2.	My morale is low here and now	❑	❑	❑	❑	❑
3.	I feel worried right now	❑	❑	❑	❑	❑
4.	I feel anxious right now	❑	❑	❑	❑	❑
5.	The only thing I can think about right now is using my smartphone	❑	❑	❑	❑	❑
6.	I miss my smartphone terribly in this moment	❑	❑	❑	❑	❑
7.	I feel an irresistible need to use my smartphone right now	❑	❑	❑	❑	❑
8.	I would like to hold my smartphone in my hand right now	❑	❑	❑	❑	❑
9.	I am irritable right now	❑	❑	❑	❑	❑
10.	I get angry easily in this moment	❑	❑	❑	❑	❑
11.	I have no patience right now	❑	❑	❑	❑	❑
12.	I feel nervous right now	❑	❑	❑	❑	❑
13.	It is difficult to think clearly right now	❑	❑	❑	❑	❑
14.	It is hard to concentrate right now	❑	❑	❑	❑	❑
15.	It is hard to focus on the task at hand right now	❑	❑	❑	❑	❑
